# Identification of Cardiovascular Risk Factors in Obese Adolescents With Metabolic Syndrome

**DOI:** 10.3389/fped.2021.745805

**Published:** 2021-10-22

**Authors:** Jung Won Lee, Young Mi Hong, Hae Soon Kim

**Affiliations:** Department of Pediatrics, Ewha Womans University School of Medicine, Seoul, South Korea

**Keywords:** metabolic syndrome, visceral obesity, left ventricular mass index, visceral fat thickness, cardiovascular disease

## Abstract

**Objective:** There are studies that show different associations between metabolic syndrome (MS) and cardiovascular disease in adolescent. This study is aimed to identify probable cardio-vascular risk factors in obese adolescents with MS.

**Methods:** Sixty-five obese adolescents with a body mass index (BMI) > 95 percentile were enrolled and divided into two groups with MS or without MS. Left ventricular mass (LVM), left ventricular mass index, ejection fraction, epicardial fat thickness, visceral fat thickness (VFT) and carotid intima-media thickness were measured. Anthropometric and blood chemistry parameters were estimated. Above parameters were compared based on presence or absence of MS.

**Results:** The prevalence of MS was 23.1% in obese adolescents. LVM showed significant correlation with body mass index (BMI), hip circumference (HC), fat mass, total cholesterol (TC), LDL-cholesterol (LDL-C) and waist circumference (WC). VFT significantly correlated with WC, BMI, hip circumflex (HC), obesity index (OI), fat %, fat mass, insulin, TC, LDL-C, insulin, triglyceride (TG), glucose, homeostatic model assessment for insulin resistance (HOMA-IR) and leptin.

**Conclusions:** Screening for the MS in overweight adolescents may help to predict risk of future cardiovascular disease. These data suggest that LVMI and VFT are significant parameters for predicting cardiovascular disease risk in obese adolescents.

## Introduction

The prevalence of adolescent metabolic syndrome (MS) has increased substantially due to lack of physical activity, unhealthy diet and lifestyle, and incomplete sleep due to presence of electrical devices.

However, there was no definition of adolescent MS until now, and most definitions have been adapted from the Adult Treatment Panel III (ATP III) definition, published by Cook et al. (1). The International Diabetes Federation (IDF) released its unique definition of MS in children and adolescents in 2007 (i.e., IDF 2007 definition), at the age of 10 yr and more, MS requires the presence of abdominal obesity (≥90th percentile) plus, the presence of two or more of elevated triglycerides (TG) (≥150 mg/dL), low high-density lipo-protein-cholesterol (HDL-C) (<40 mg/dL), high blood pressure (Systolic blood pressure ≥130 or diastolic blood pressure ≥85 mm Hg), and elevated plasma glucose (Fasting glucose ≥100 mg/dL or known type2 DM) (2).

Visceral fat and insulin resistance are key factors of MS development (3). The visceral fat accumulation is associated with vascular atherosclerosis and cardiovascular outcome after all (4). These consequences are from pro-inflammatory cytokines originated from visceral fat tissue (5). For example, epicardial fat around heart interacts with myocardium and coronary arteries through cytokines (6). Thus, definition of MS has expanded to include many inflammatory cytokines (7). It has been postulated that obesity dysregulates blood adipokine levels, may induce insulin resistance, and finally influence the development of MS and vascular complications (8).

Although increased number of metabolic risk factors and their complex interactions contribute to the occurrence of MS, this has not been well-studied in adolescent populations (9).

The aim of this study was to identify specific risk factors from visceral obesity closely related to future cardiovascular consequence in obese adolescent populations.

## Methods

### Study Participants

Sixty five obese adolescents (31 males, 34 females) aged between 15 and 17 in a high school participated in this study. They were divided into two groups: the MS group (total = 15, male = 9, female = 6) and the group without MS (total = 50, male = 22, female = 28). We measured MS in adolescents using a modified cook's definition. Adolescent obesity was defined as body mass index (BMI) above the 95th percentile for age and sex by the survey of Korean Centers of Disease Control and Prevention in 2007 (10). Adolescents with other endocrine disease, psychiatric problem or other severe and chronic disease were excluded. Informed consents were obtained from the parents or guardians. The study was conducted according to the guidelines of the Declaration of Helsinki, and approved by Ethical Committee of Ewha Womans University, College of Medicine, Seoul, Korea (approval No: 0319/12.06.2020).

### Anthropometric Measurement

Anthropometric data of weight, height, waist circumference (WC), BMI, obesity index (OI), hip circumflex (HC), fat mass, fat percentage and blood pressure (BP) from both groups were obtained. Both the height and weight were taken with light dressed and shoes were off on the height measuring instrument (JENIX, Samhwa Co., Ltd., Seoul, Korea), and the eyes were straightened. The vertical distance from the soles of the feet to the top of the head was measured and recorded up to 0.1 cm. The weight was recorded up to 0.1 kg. WC was measured at the mid-point between 12th rib and the mid portion of the superior iliac crest. BMI was calculated by dividing the body weight (kilogram) by height (meter) in meter square. OI was obtained by the equation using the standard weight value corresponding to the 50th percentile of the weight data chart for Korean children. HC was measured from the most protruding part of the hip. Fat mass and fat percentage were measured by bioelectric impedance analysis (BIA) (InBody 720, Biospace Co., Ltd., Seoul, Korea). BIA can be used as an alternative to gold standard dual-energy X-ray absorptiometry (DXA) admitting BIA underestimate body fat percentage values compared to DXA (11). BP was measured after 10 min of bed rest using an automatic oscillometric BP device in a supine position (Omron HEM-7320-LA, Omron Healthcare Co. Ltd, Kyoto, Japan).

### Laboratory Measurement

Blood was taken from all 65 subjects after 14 h fasting. Blood glucose, total cholesterol, TG, low density lipoprotein-cholesterol (LDL-C), HDL-C, aspartate aminotransferase (AST), alanine aminotransferase (ALT), Serum TNF-α and IL-6 levels were measured by sandwich enzyme immunoassay-based Quantikine Human interleukine and TNF-α kit (R&D system Inc., Minneapolis, MN, USA). Serum leptin, adiponectin and insulin levels were measured using the Human Leptin 125 tubes radioimmunoassay kit (Linco Resarch Inc., St. Charles, MO, USA), radioimmunoassay human adiponectin 125 tubes radioimmunoas-say kit (Linco Research, Inc. St. Charles, MO, USA) and human insulin chemiluminescence immunoassay kit (insulin, Siemens Centaur, Holliston, MA, USA), respectively.

Insulin resistance was measured by the homeostasis model assessment of insulin resistance (HOMA-IR), by dividing the multiple of insulin (μU/mL) and serum glucose (mmol/L).

### Carotid Artery Ultrasound Measurement

Carotid intima-media thickness (CIMT) was measured with neck ultrasound iU22 (Intelligent Ultrasound System, Philips Medical System, Amsterdam, The Netherlands). After the subjects lied down and rest for at least 30 min, probe was placed on common carotid artery 1 cm proximal from the carotid bifurcation. Three points of thickest intima-media lengths were measured and average was recorded.

### Abdominal Ultrasonography Measurement

Abdominal fat thickness including subcutaneous fat thickness, visceral fat thickness and pre-peritoneal fat thickness were measured with abdominal ultrasound Acuson XP128 (Acuson, Mountain View, CA, USA) by placing a 3.5 MHz linear-array probe 1 cm above the navel at end-expiratory phase. Subcutaneous fat thickness was measured from skin to anterior abdominal muscle, visceral fat thickness from posterior abdominal muscle to anterior wall of aorta, and pre-peritoneal fat thickness from the subcutaneous layer to the peritoneum, respectively. Same experienced radiologist performed the measurement.

### Echocardiographic Parameters Measurement

Echocardiographic parameters such as epicardial fat thickness (EFT), LV dimension, and systolic/diastolic parameters were estimated by echocardiography (Acuson Sequoia-C 512, Siemens, CA, USA). The subjects lied down in left lateral decubitus with using phased-array echocardiograms in M-mode, 2D, and pulsed and color-flow Doppler settings. EFT on the free wall of right ventricle was measured in parasternal long axis and short axis views. LV dimension was measured by perpendicular to the long axis of the left ventricle. Systolic and diastolic parameters such as stroke volume (SV) was determined by the aortic annular cross-sectional area multiplied by the aortic time-velocity integral. Cardiac output (CO) was calculated by multiplying SV and the heart rate (HR). Ejection fraction (EF) was calculated from biplane Simpson formula and fractional shortening (FS) was calculated using left ventricular internal dimensions. Early diastolic (E), late atrial (A) peak velocities, E/A ratios, and E-wave deceleration time (ms) were measured from pulsed doppler mode in apical window. Left ventricular mass (LVM) was calculated calculated from American Society of Echocardiography guideline, and left ventricular mass index (LVMI) was calculated from LMV devided by body surface area (Monstella method).

LVM (g) = 0.8 [1.04 (STd + LVIDd + PWTd)3 – LVIDd3] + 0.6 g

□ STd = interventricular septal thickness at end-diastole (mm)
□ LVIDd = LV end-diastolic dimension (mm)
□ PWTd = posterior wall thickness at end-diastole (mm)

Body surface area: {[Ht (cm) × Bwt (kg)]/3,600}^1/2^

□ Ht: Height Bwt; body weight.

### Brachial-Ankle Pulse Wave Velocity and Ankle Brachial Index Measurement

Brachial-ankle pulse wave velocity (baPWV) were measured using a volume-plethysmographic apparatus (Colin Co. Ltd., Komaki, Japan). The subject was examined in supine position for at least 5 min with taking their usual medication. The device gathered oscillometric waveforms and simultaneously calculates the time intervals between brachium and ankle waveforms. Two sets of baPWV values were measured and left and right data were showed. Ankle Brachial index (ABI) was calculated as the ratio of systolic BP in ipsilateral ankle and brachium.

### Statistical Analysis

We performed statistical analyses using Statistical Package for the Social Sciences (SPSS) (version 20, SPSS Inc. Chicago, IL, USA). Data were presented as means and standard deviations. The comparison of continuous variables was done using the Student *t*-test or two-way ANOVA. Univariate and multivariate logistic regression models were implemented to examine the independent relationship between the dependent variable MS and other variables of metabolic parameters. *P*-value < 0.05 was considered as statistically significant.

## Results

### The Prevalence of Metabolic Syndrome and Clustering of Metabolic Components

Of the total of sixty-five subjects (31 males, 34 females), 23.1% (15/65) showed an incidence of MS that satisfied more than three of the criteria of MS.

The percentage of the subjects meeting MS criteria were WC ≥ 90 percentile (52.3%), systolic BP or diastolic BP ≥ 90 percentile (mmHg) (52.3%), and HDL- C ≤ 40 (mg/dL) (46.2%), followed by TG ≥ 110(mg/dL) (26.2%) and fasting glucose ≥ 100 (mg/dL) (3.1%) in Table 1.

**Table 1:** Prevalence of components of MS in obese adolescents.

**Criteria**	***N*** **(M/F)**	**%**
WC ≥ 90 percentile	34(17/17)	52.3
SBP or DBP ≥ 90 percentile(mmHg)	34(13/21)	52.3
HDL-C < 40 (mg/dL)	30(17/13)	46.2
Triglyceride ≥ 110 (mg/dL)	17(11/6)	26.2
Fasting glucose ≥ 100 (mg/dL)	2(1/1)	3.1

*MS, metabolic syndrome; WC, waist circumference; SBP, systolic blood pressure; DBP, diastolic blood pressure; HDL-C, high density lipotrotein-cholesterol; N, number; %, percent from total subjects*.

### Comparison of Anthropometric Data

There was no difference in anthropometric data between MS and non-MS groups of both females and males in Table 2.

**Table 2:** Comparison of anthropometric data in obese adolescents.

	**Male (***n*** = 31)**			**Female (***n*** = 34)**		
	**MS(–) (***n*** = 22)**	**MS(+) (***n*** = 9)**	***P***	**MS(–) (***n*** = 28)**	**MS(+) (***n*** = 6)**	***P***
Age (year)	16.4 ± 0.9	16.3 ± 0.9	0.812	16.6 ± 0.8	16.5 ± 0.8	0.649
Height (cm)	171.1 ± 5.3	174.6 ± 3.9	0.089	159.2 ± 4.7	160.0 ± 5.2	0.74
Weight (kg)	84.3 ± 16.8	94.0 ± 13.8	0.139	70.0 ± 6.4	76.8 ± 14.2	0.297
BMI (kg/m^2^)	28.6 ± 4.63	30.7 ± 3.8	0.248	27.5 ± 1.8	29.9 ± 4.2	0.234
HC (cm)	90.5 ± 11.3	97.5 ± 7.8	0.102	78.6 ± 4.2	84.9 ± 7.1	0.082
WC (cm)	103.8 ± 7.9	109.0 ± 9.2	0.124	99.3 ± 4.6	104.4 ± 9.1	0.048
WH-R	0.86 ± 0.07	0.89 ± 0.03	0.11	0.79 ± 0.04	0.82 ± 0.04	0.152
OI (%)	131.0 ± 20.5	139.3 ± 17.1	0.29	132.3 ± 9.4	143.1 ± 18.6	0.221
Fat (%)	31.7 ± 6.3	33.0 ± 5.3	0.586	41.6 ± 3.7	42.1 ± 4.5	0.778
Fat mass (kg)	27.6 ± 12.0	31.6 ± 9.0	0.376	29.6 ± 3.9	32.6 ± 7.9	0.195
SBP(mm Hg)	124.2 ± 13.3	129.3 ± 18.2	0.39	114.8 ± 8.4	118.8 ± 12.6	0.35
DBP(mm Hg)	76.1 ± 7.6	82.6 ± 16.2	0.277	71.0 ± 6.0	76.3 ± 8.3	0.078

*MS, metabolic syndrome; BMI, body mass index; HC, hip circumference; WC, waist circumference; WH-R, waist hip ratio; OI, obesity index; SBP, systolic blood pressure; DBP, diastolic blood pressure*.

### Comparison of Biochemistry Parameters

LDL-C was significantly increased in MS females (131.1 ± 19.5 vs. 96.7 ± 27.5 mg/dL, *p* = 0.007), TG was significantly increased and HDL-C was significantly decreased in both MS males and MS females than control group. adolescents without MS. Adiponectin and insulin like growth factor binding protein 3 (IGFBP-3) were significantly decreased in MS females compared to non-MS females in Table 3.

**Table 3:** Comparison of biochemistry parameters in obese adolescents.

	**Male (***n*** = 31)**			**Female (***n*** = 34)**		
	**MS(–) (***n*** = 22)**	**MS(+) (***n*** = 9)**	***P***	**MS(–) (***n*** = 28)**	**MS(+) (***n*** = 6)**	***P***
Glucose(mg/dL)	91.3 ± 7.3	86.2 ± 7.1	0.089	85.7 ± 4.4	89.3 ± 10.2	0.433
Insulin (mU/L)	17.1 ± 9.1	18.9 ± 10.4	0.667	13.0 ± 5.7	24.1 ± 21.5	0.265
HOMA-IR	3.8 ± 1.8	4.0 ± 2.3	0.742	2.7 ± 1.2	5.6 ± 5.8	0.277
TC (mg/dL)	161.7 ± 33.8	152.7 ± 36.5	0.517	165.8 ± 31.1	196.6 ± 24.3	0.031
LDL-C (mg/dL)	99.9 ± 26.2	91.3 ± 30.1	0.433	96.7 ± 27.5	131.1 ± 19.5	0.007
TG (mg/dL)	81.4 ± 36.4	145.3 ± 60.9	0.001	61.6 ± 26.8	151.8 ± 36.1	0.001
HDL-C (mg/dL)	51.0 ± 6.8	45.5 ± 2.6	0.003	59.4 ± 9.8	44.6 ± 2.6	0.000
AST (IU/L)	22.2 ± 6.8	39.1 ± 22.5	0.056	22.0 ± 6.4	30.3 ± 30.2	0.533
ALT (IU/L)	29.9 ± 16.0	52.7 ± 34.2	0.086	17.5 ± 10.1	47.5 ± 65.1	0.313
hs-CRP (mg/L)	1.4 ± 2.1	1.2 ± 2.0	0.828	1.6 ± 2.0	1.5 ± 1.3	0.888
IL-6 (pg/mL)	6.5 ± 3.3	4.9 ± 1.7	0.21	7.1 ± 7.1	6.2 ± 1.4	0.782
TNF-a (pg/mL)	3.4 ± 2.5	3.0 ± 2.2	0.804	31.4 ± 77.5	1.7 ± 2.2	0.537
Leptin (ug/L)	8.3 ± 4.7	9.7 ± 4.6	0.486	17.4 ± 6.5	18.9 ± 3.5	0.626
Adiponectin(ng/mL)	7379.5 ± 4789.6	6788.3 ± 2960.4	0.751	8514.3 ± 2938.4	5469.4 ± 1054.2	0.001
FFA (uEq/L)	561.6 ± 294.5	660.8 ± 358.4	0.434	655.3 ± 429.4	777.1 ± 174.3	0.504
IGFBP-3 (ng/mL)	3782.7 ± 508.7	3577.3 ± 413.4	0.295	3598.7 ± 491.3	4108.3 ± 258.4	0.02

*MS, metabolic syndrome; HOMA-IR, homeostasis model assessment of insulin resistance; AST, aspartate aminotransferase; ALT, alanine aminotransferase; TC, total cholesterol; LDL-C, low density lipotrotein cholesterol; TG, triglyceride; HDL-C, high density lipotrotein cholesterol; hs-CRP, high sensitive C reactive protein; IL-6, interleukin-6; TNF, tumor necrosis factor; FFA, free fatty acid; IGFBP, insulin growth factor binding protein*.

### Comparison of the Geometrical Analysis of the Carotid Artery

The geometrical analysis of the carotid artery had no differential data including intimal media thickness between two groups of both females and males in Table 4.

**Table 4:** Comparison of geometrical parameters of carotid artery in the obese adolescents.

	**Male (***n*** = 31)**			**Female (***n*** = 34)**		
	**MS(–) (***n*** = 22)**	**MS(+) (***n*** = 9)**	***P***	**MS(–) (***n*** = 28)**	**MS(+) (***n*** = 6)**	***P***
IMT (mm)	0.6 ± 0.2	0.6 ± 0.1	0.67	0.5 ± 0.1	0.6 ± 0.2	0.506
S diameter (mm)	6.4 ± 0.1	6.4 ± 0.7	0.827	5.3 ± 0.5	5.3 ± 0.5	0.997
D diameter (mm)	6.5 ± 0.7	13.8 ± 21.5	0.367	6.0 ± 0.5	17.2 ± 24.5	0.366
LCSA (m^2^)	28.7 ± 7.0	28.8 ± 4.2	0.986	22.3 ± 4.0	22.5 ± 4.1	0.895
WCSA (m^2^)	12.4 ± 6.2	11.5 ± 3.3	0.689	9.3 ± 2.5	10.9 ± 4.8	0.284
CSC	0.2 ± 0.1	0.1 ± 0.1	0.207	0.1 ± 0.1	0.2 ± 0.1	0.179
CSD	0.01 ± 0.00	0.00 ± 0.00	0.183	0.01 ± 0.00	0.01 ± 0.00	0.208

*MS, metabolic syndrome; IMT, intimal medial thickness; S diameter, carotid artery systolic diameter; D diameter, carotid artery diastolic diameter; LCSA, Lumen cross-sectional area; WCSA, wall cross-sectional area; CSC, cross-sectional compliance; CSD, cross-sectional distensibility*.

### Comparison of Abdominal Sonographic Data

In the abdominal sonographic data, VFT was significantly higher in MS females compared to non-MS females (42.0 ± 7.5 vs. 31.1 ± 6.9 mm, *p* = 0.004) in Table 5.

**Table 5:** Comparison of abdominal sonographic data in the obese adolescents.

	**Total (***n*** = 65)**			**Male (***n*** = 31)**		**Female (***n*** = 34)**		
	**MS (–)** **(***n*** = 50)**	**MS(+)** **(***n*** = 15)**	***p***	**MS(–)** **(***n*** = 22)**	**MS(+)** **(***n*** = 9)**	**MS(–)** **(***n*** = 28)**	**MS(+)** **(***n*** = 6)**	***P***
VFT (mm)	34.6 ± 13.1	38.3 ± 12.7	0.038	37.9 ± 17.9	37.4 ± 20.2	31.1 ± 6.9	42.0 ± 7.5	0.034
SFT (mm)	20.5 ± 6.9	21.2 ± 4.8	0.595	21.4 ± 7.8	19.9 ± 5.7	19.4 ± 5.5	23.2 ± 2.3	0.455
PFT (mm)	12.8 ± 3.9	12.9 ± 3.4	0.996	10.9 ± 4.5	13.7 ± 4.8	14.9 ± 3.8	12.4 ± 2.3	0.957

*VFT, visceral fat tissue; SFT, subcutaneous fat tissue; PFT, properitoneal fat tissue*.

### Comparison of Echocardiographic Data

In the echocardiography data including epicardial fat thickness, there was significant increase in left ventricular mass (LVM) (205.9 ± 44.5 vs. 258.7 ± 59.0 g, *p* = 0.008) and left ventricular mass index (LVMI) (1.9 ± 0.4 vs. 2.4 ± 0.5 g/m^2^, *p* = 0.047) in MS (+) males in Table 6.

**Table 6:** Comparison of echocardiographic data in the obese adolescents.

	**Total (***n*** = 65)**			**Male (***n*** = 31)**		**Female (***n*** = 34)**		
	**MS(–)** **(***n*** = 50)**	**MS(+)** **(***n*** = 15)**	***P***	**MS(–)** **(***n*** = 22)**	**MS(+)** **(***n*** = 9)**	**MS(–)** **(***n*** = 28)**	**MS(+)** **(***n*** = 6)**	***P***
SV (mL)	63.5 ± 9.8	59.5 ± 14.4	0.065	66.7 ± 10.2	62.4 ± 9.4	61.8 ± 19.8	56.3 ± 11.5	0.767
CO (L)	4.7 ± 1.1	4.2 ± 0.9	0.065	4.7 ± 0.7	4.3 ± 1.0	4.7 ± 1.6	4.2 ± 0.8	0.631
IVS (mm)	1.0 ± 0.7	0.8 ± 0.6	0.167	0.9 ± 0.1	0.9 ± 0.1	1.0 ± 1.3	0.8 ± 0.1	0.265
PWT (mm)	0.9 ± 0.1	0.9 ± 0.1	0.725	1.0 ± 0.1	1.0 ± 0.1	0.8 ± 0.1	0.8 ± 0.1	0.999
EF (%)	61.3 ± 6.0	60.5 ± 3.8	0.465	59.5 ± 7.0	61.0 ± 3.7	62.8 ± 5.5	59.4 ± 3.7	0.595
FS (%)	32.8 ± 4.5	32.3 ± 2.8	0.74	32.3 ± 3.5	32.9 ± 2.7	33.9 ± 4.8	31.4 ± 2.9	0.104
LVM (g)	176 ± 36.7	211.6 ± 45.9	0.012	205.9 ± 44.5	258.7 ± 59.0	141.3 ± 21.0	158.0 ± 36.7	0.001
LVMI (g/m^2^)	1.8 ± 0.7	2.2 ± 0.33	0.065	1.9 ± 0.4	2.4 ± 0.5	1.6 ± 0.2	1.9 ± 0.34	0.053
MVE (m/sec)	0.99 ± 0.2	0.93 ± 0.3	0.678	1.0 ± 0.2	0.9 ± 0.2	0.99 ± 0.2	0.95 ± 0.2	0.567
MVA(m/sec)	0.5 ± 0.1	0.5 ± 0.1	0.225	0.5 ± 0.1	0.5 ± 0.1	0.5 ± 0.1	0.5 ± 0.1	0.997
MVE/A	1.9 ± 0.6	2.0 ± 0.6	0.559	1.8 ± 0.4	2.1 ± 0.6	2.0 ± 0.5	2.0 ± 0.6	0.273
MVE-DT (ms)	152.6 ± 25.7	145.3 ± 36.8	0.381	159.7 ± 28.8	147.3 ± 36.7	148.3 ± 21.8	141.8 ± 36.8	0.886
EFT (PSAX) (mm)	0.2 ± 0.2	0.2 ± 0.04	0.68	0.2 ± 0.1	0.2 ± 0.03	0.2 ± 0.02	0.2 ± 0.04	0.978

*MS, metabolic syndrome; SV, stroke volume; CO, cardiac output; IVS, interventricular septal thickness; PWT, posterior wall thickness; EF, ejection fraction; FS, fraction shortening; LVM, left ventricle Mass; LVMI, left ventricle mass index; E, early diastolic velocity; A, late atrial peak velocity; DT, deceleration time; EFT (PSAX), epicardial fat thickness at parasternal short axis view*.

### Comparison of Pulse Wave Velocity and Ankle Brachial Index

There was significant increase of left ABI in the MS (+) females (106 ± 6.8 vs. 100.4 ± 7.9, *p* = 0.021). However, no significant difference was observed in heart rate, RbaPWV, LbaPWV and right ABI between the MS (–) and MS (+) groups in Table 7.

**Table 7:** Comparison of pulse wave velocity and ankle brachial index in obese adolescents.

	**Male (***n*** = 31)**			**Female (***n*** = 34)**		
	**Ms(–) (***n*** = 22)**	**MS(+) (***n*** = 9)**	***P***	**MS(–) (***n*** = 28)**	**MS(+) (***n*** = 6)**	***P***
HR (/min)	73.4 ± 8.4	70.6 ± 9.2	0.323	70.6 ± 9.1	75.2 ± 10.1	0.134
RbaPWV (cm/s)	1102.8 ± 120.1	1117.8 ± 138.2	0.639	931.5 ± 92.8	946.5 ± 100.4	0.401
LbaPWV (cm/s)	1130.2 ± 115.1	1112.0 ± 143.2	0.843	913.3 ± 101.2	952.1 ± 103.2	0.221
RABI	102.8 ± 7.9	105.1 ± 6.5	0.321	105.8 ± 7.2	101.7 ± 6.8	0.088
LABI	104.1 ± 6.7	106.7 ± 5.4	0.601	106.5 ± 6.8	100.4 ± 7.9	0.021

*MS, metabolic syndrome; HR, Heart rate; RbaPWV, right brachial ankle pulse wave velocity; LbaPWV, left brachial ankle pulse wave velocity; RBAI, right brachial ankle index; LBAI, left brachial ankle index*.

### Correlation Between the LV Mass and Other Metabolic Parameters

LV mass is significantly associated with BMI, HC, fat mass, insulin, TC, LDL-C, and WC (*P* < 0.05) in Table 8.

**Table 8:** Correlation between LV mass and other metabolic parameters.

	**Estimate**	**SE**	**Lower CL**	**Upper CL**	***P***
BMI (kg/m^2^)	4.385	1.244	1.883	6.886	0.009
HC (cm)	2.121	0.639	0.835	3.406	0.017
Fat mass (kg)	1.767	0.543	0.673	2.861	0.022
OI (%)	0.922	0.287	0.343	1.5	0.052
Insulin (mU/L)	1.261	0.442	0.371	2.15	0.046
HOMA-IR	5.06	1.841	1.359	8.762	0.084
TC (mg/dL)	0.386	0.146	0.092	0.68	0.022
LDL-C (mg/dL)	0.397	0.168	0.059	0.734	0.012
WC (cm)	1.298	0.576	0.141	2.455	0.028

*BMI, body mass index; HC, hip circumference; OI, obesity index; HOMA-IR, homeostasis model assessment of insulin resistance; TC, total cholesterol; WC, waist circumference; LDL –C, low density lipoprotein-cholesterol*.

### Correlation Between Visceral Fat Tissue and Other Metabolic Parameters

Visceral fat tissue is significantly associated with WC, DBP, TG, LDL-C, TC, glucose, BMI, HC, waist-hip ratio (WHR), OI, Fat mass, insulin, HOMA-IR, and leptin in Table 9.

**Table 9:** Correlations between visceral fat tissue and other metabolic parameters.

**Variable**	**Estimate**	**StdErr**	**LowerCL**	**UpperCL**	***P***
WC (cm)	1.074	0.19	0.692	1.455	<0.001
DBP (mm Hg)	0.476	0.205	0.065	0.887	0.024
TG (mg/dL)	0.118	0.041	0.036	0.199	0.006
LDL-C (mg/dL)	0.203	0.065	0.072	0.335	0.003
TC (mg/dL)	0.199	0.056	0.086	0.313	0.001
Glucose (mg/dL)	−0.59	0.285	−1.163	−0.018	0.044
BMI (kg/m^2^)	2.537	0.43	1.673	3.401	<0.0001
HC (cm)	1.208	0.228	0.75	1.666	<0.0001
WHR	98.053	45.313	6.945	189.161	0.036
OI	0.553	0.101	0.35	0.756	<0.0001
Fat %	1.743	0.388	0.962	2.524	<0.0001
Fat mass (kg)	1.138	0.179	0.777	1.498	<0.0001
Insulin (mU/L)	0.548	0.177	0.193	0.903	0.003
HOMA-IR	1.958	0.75	0.451	3.466	0.012
Leptin (ug/L)	0.792	0.381	0.024	1.56	0.043

*WC, waist circumference; DBP, diastolic blood pressure; TG, triglyceride; LDL-C, low density lipotrotein-cholesterol; TC, total cholesterol; BMI, body mass index; HC, hip circumference; WHR, waist hip ratio; OI, obesity index; HOMA-IR, homeostasis model assessment of insulin resistance*.

## Discussion

Given that there is no well-established uniform definition of childhood MS (1), most frequently used definitions have been adopted from the Adult Treatment Panel III (ATP III) definition, modified and adapted by Cook et al. (12). And the IDF made the definition of MS in children ≥ 10 years of age in 2007 to find the global prevalence of MS. These two measurement definitions had different cut off levels for hyperglyceridemia and elevated blood pressure. In this study of MS in obese adolescents, we applied modified Cook's definitions of the ATP III definitions.

Prevalence of MS in adolescent in representative studies was 28.7% in the study of cook et al. (12). In this study, we found the prevalence of MS in obese children was 23.1% (15/65) similar to previous studies.

It seems appropriate to apply the same factors from adults in establishing a definition of MS in adolescent, such as the dyslipidemia, hypertension, and obesity which tend to track into adulthood (12). But some components need further correction in adolescents. For instance, impaired fasting glucose is very low but impaired glucose tolerance is fairly common in childhood (13, 14). So fasting glucose threshold need to be lowered and impaired glucose tolerance should be proposed as components of the MS (15). The cut off level for fasting glucose it our study is 110 mg/dL, which is lower than that of ATPIII. Aside from this, the pubertal status, age range, ethnicity need to be considered in defining MS of adolescents (1, 7).

Visceral fat is usually measured by WC or other imaging methods. Among the parameters of MS, WC is easy and practical for screening children at risk of MS (2). Although WC is a better marker and widely used parameter of abdominal adipose tissue accumulation than the BMI, the increased WC is not sufficient to define visceral obesity alone, because of increased subcutaneous fat (16). In this study, we used abdominal sonographic method to estimate the visceral fat and found that VFT had significant correlation with WC.

The accumulation of visceral fat contrary to abdominal subcutaneous fat plays a significant role in the pathophysiology of the MS with insulin resistance in adolescent (17, 18). Visceral fat accumulation affects the adipose tissue to over secret leptin and hyposecretion of adiponectin (14).

Leptins correlate with BMI in children. High plasma leptin level is related to the development of essential hypertension, hyperinsulinemia and dyslipidemia (19). Adiponectin originates mainly from adipocytes and is an adipocytokine that is acting against atherogenic, diabetic and pro-inflammatory effect (8). Decreased adiponectin levels are associated with dysmetabolic state and cause MS. Furthermore, visceral obesity may lead to ectopic fat deposition in skeletal muscle, liver, heart, etc. (16). In this study, there was increased VFT in both MS male and MS female compared to control.

EFT at right ventricle, left ventricular apex and atrium seems to correlate with visceral fat and act as cardiometabolic risk factor (20). Measuring echocardiographic epicardial fat tissue can be used as a method for quantification of visceral fat. The epicardial adipose tissue contact and interact with the myocardial tissue and coronary arteries by secreting pro-inflammatory cytokines and vasoactive peptides (6). These molecules can develop glucose intolerance and atherosclerosis and increase cardiovascular risk (6, 21). In our study, we found an increased EFT both in MS males and MS females.

Obesity and MS demand increased metabolic and blood supply due to greater adipose tissue, which increase blood volume and cardiac preload. And vascular alterations such as arterial stiffness and peripheral resistance increase afterload to the heart. Finally, they incur LV hypertrophy and LV diastolic dysfunction (22, 23). In our study, there was significant increase in LVM and LVMI in MS male. MS female also showed some trend of increased LVM without significance. In short, this study found VFT and LVM is significantly correlated with metabolic and cardiac parameters. So, screening for the MS in overweight adolescents may help to predict future cardiovascular disease.

As CVD is mainly result of atherosclerosis, the predictors of atherosclerosis are important. And CIMT is a highly predictive marker of progression of atherosclerosis in patients with MS (24). However, we could not find abnormal CIMT from both MS and non-MS groups in this study. This might be due to young age groups whose peripheral artery is very resistant to local vascular inflammation and atherosclerosis progression. Also, baPWV data from our study was not efficient either. In our study, female LABI only showed statistically significant difference. The reason why there was no statistical difference between the remaining data is believed to be due to the low accuracy of the test. Because baPWV method include peripheral vessels, it is less accurate and does not reflect the degree of arteriosclerosis in the central blood vessels, but rather changes in peripheral arteries can be reflected. Carotid-femoral PWV (cfPWV) is better alternative since it predicted cardiovascular mortality (25). However, femoral artery waveforms in cfPWV measure are difficult to accurately record in metabolic syndrome, obesity, diabetes, peripheral artery patients. So, we suggest other non-invasive and high accuracy central blood pressure measurements such as radial tonometry or brachial cuff pulse volume plethysmography for future research (26).

Previous studies of MS in adolescents showed abundant evidence of risk factors for CVD (18). For example, autopsy studies in childhood showed that obesity, high BP, high TG, and low HDL-C are related to coronary atherosclerosis (27, 28). So, early identification of these risk factors is important in chronic disease prevention (29, 30). In the present study, we found significant differences in VFT and LVM in obese adolescents with MS.

Hyperinsulinemia and insulin resistance are well-known risk factors for CVD. Hyperinsulinemia directly contributes to deteriorated renal function and increased risk of CVD (31). The foremost mechanism is an up-regulation of the renin-angiotensin system, which contribute to begin hypertension, heart failure and atherosclerosis (32). These hyperinsuliemia, insulin resistance and finally upregulated renin-angiotensin system, are thought to contribute to endothelial dysfunction, atherosclerosis and CVD (32). Another story of insulin dysregulation is insulin resistance syndrome (IRS). IRS comprise of hyperinsulinemia, dyslipidemia, hypertension, and obesity. A child whose parents have IRS have greater insulin resistance and CVD risks compared with those who do not (33). So, IRS in adolescent is of potential importance, because of the high prevalence of impaired glucose tolerance and obesity, and the increasing incidence of type 2 diabetes in adolescent (34).

Adolescent obesity-induced other metabolic complication is hyperuricemia. In our study, we did not collect uric acid levels. But uric acid is associated with cardiovascular, renal and metabolic diseases. It is because serum uric acid (SUA) is known as an antioxidant factor in the extracellular space, but as a pro-oxidant factor inside the cell (35).

Among these diseases, the link between SUA levels and hypertension seem to be the strongest. When children with high SUA were followed for 10 years, high incidence of MS in male subjects are observed (36). Also increased SUA is associated with new-onset primary hypertension in children (37). It is noted that hypertensive children with elevated SUA have a higher prevalence of obesity-related CVD (38). And one observational cohort study shows uric acid elevation is commonly found in hypertriglyceridemia, obesity, insulin resistance and hypertension. But they failed to show low uric acid level in well-controlled hypertension patients. Until now, whether elevated uric acid level actually causes CVD or increase MS remains to be confirmed (39).

There are several limitations to this study. First, the study included small cohort not enough to analyze all the data for each gender. Further research with large adolescent population is inevitable. Second, there is absence of follow up data. Third, there is lack of comprehensive assessment according to diverse adolescent age group, because the age range is confined to 15–18. Finally, even though measurements were conducted by same sonographer, there could be some intraobserver variability of reproducibility of measuring the parameters such as IMT, VFT, and EFT due to the sonographer's subjectivity. This could have been avoided by performing three measurements and averaging them.

Since the time between adolescent obesity and occurrence of clinical disease is relatively short, longitudinal prospective design dealing from adolescence to adulthood is needed to explore the complexity of interrelation of MS risk factors. The prospective study of longer time course may offer a better opportunity in adolescent obesity and CVD risk research.

Overall, We defined adolescent MS as to meet more than 3 categories (WC ≥ 90th percentile, BP ≥ 90th percentile, TG ≥ 110 mg/dL, HDL ≤ 40 mg/dL, fasting glucose ≥ 110 mg/dL). The study subjects are obese male and female adolescents defined as BMI above the 95th percentile for age and sex by the survey of Korean Centers of Disease Control and Prevention in 2007. In this setting, we found both VFT in inn total and female MS group and LVM in total and male adolescents were increased. Although there were statistical differences between male and female adolescents, it is meaningful that there were differences in VFT and LVM between the entire MS and non-MS groups.

In conclusion, one can assume that VFT and LVM are appropriate and possible markers for predicting increased future cardiovascular risk in obese adolescents.

## Data Availability Statement

The raw data supporting the conclusions of this article will be made available by the authors, without undue reservation.

## Ethics Statement

The studies involving human participants were reviewed and approved by IRB at Ewha Womans University (Approval No: 0319/12/06.2020). Written informed consent to participate in this study was provided by the participants' legal guardian/next of kin.

## Author Contributions

JL, YH, and HK: conceptualization. JL: methodology, formal analysis, data curation, writing—original draft, and supervision. HK: investigation. YH and HK: writing—review and editing. All authors contributed to the article and approved the submitted version.

## Conflict of Interest

The authors declare that the research was conducted in the absence of any commercial or financial relationships that could be construed as a potential conflict of interest.

## Publisher's Note

All claims expressed in this article are solely those of the authors and do not necessarily represent those of their affiliated organizations, or those of the publisher, the editors and the reviewers. Any product that may be evaluated in this article, or claim that may be made by its manufacturer, is not guaranteed or endorsed by the publisher.
